# Previous disorders and depression outcomes in individuals with 12-month major depressive disorder in the World Mental Health surveys

**DOI:** 10.1017/S2045796021000573

**Published:** 2021-11-11

**Authors:** Annelieke M. Roest, Ymkje Anna de Vries, Ali Al-Hamzawi, Jordi Alonso, Olatunde O. Ayinde, Ronny Bruffaerts, Brendan Bunting, José Miguel Caldas de Almeida, Giovanni de Girolamo, Louisa Degenhardt, Silvia Florescu, Oye Gureje, Josep Maria Haro, Chiyi Hu, Elie G. Karam, Andrzej Kiejna, Viviane Kovess-Masfety, Sing Lee, John J. McGrath, Maria Elena Medina-Mora, Fernando Navarro-Mateu, Daisuke Nishi, Marina Piazza, José Posada-Villa, Kate M. Scott, Juan Carlos Stagnaro, Dan J. Stein, Yolanda Torres, Maria Carmen Viana, Zahari Zarkov, Ronald C. Kessler, Peter de Jonge

**Affiliations:** 1 Department of Developmental Psychology, University of Groningen, Groningen, The Netherlands; 2 College of Medicine, Al-Qadisiya University, Diwaniya Governorate, Iraq; 3 Health Services Research Unit, IMIM-Hospital del Mar Medical Research Institute, Barcelona, Spain; 4 CIBER en Epidemiología y Salud Pública (CIBERESP), Spain; 5 Pompeu Fabra University (UPF), Barcelona, Spain; 6 Department of Psychiatry, University of Ibadan, Ibadan, Nigeria; 7 Universitair Psychiatrisch Centrum – Katholieke Universiteit Leuven (UPC-KUL), Campus Gasthuisberg, Leuven, Belgium; 8 School of Psychology, Ulster University, Londonderry, UK; 9 Lisbon Institute of Global Mental Health and Chronic Diseases Research Center (CEDOC), NOVA Medical School|Faculdade de Ciências Médicas, Universidade Nova de Lisboa, Lisbon, Portugal; 10 IRCCS Istituto Centro San Giovanni di Dio Fatebenefratelli, Brescia, Italy; 11 National Drug and Alcohol Research Centre, University of New South Wales, Sydney, Australia; 12 National School of Public Health, Management and Development, Bucharest, Romania; 13 Department of Psychiatry, University College Hospital, Ibadan, Nigeria; 14 Parc Sanitari Sant Joan de Déu, CIBERSAM, Universitat de Barcelona, Sant Boi de Llobregat, Barcelona, Spain; 15 Shenzhen Institute of Mental Health & Shenzhen Kangning Hospital, Shenzhen, China; 16 Department of Psychiatry and Clinical Psychology, St George Hospital University Medical Center, Beirut, Lebanon; 17 Balamand University, Faculty of Medicine, Beirut, Lebanon; 18 Institute for Development, Research, Advocacy and Applied Care (IDRAAC), Beirut, Lebanon; 19 WSB University, Torun, Poland; 20 University of Lower Silesia, Wroclaw, Poland; 21 Ecole des Hautes Etudes en Santé Publique (EHESP), EA 4057, Paris Descartes University, Paris, France; 22 Department of Psychiatry, Chinese University of Hong Kong, Tai Po, Hong Kong; 23 Queensland Centre for Mental Health Research, The Park Centre for Mental Health, Wacol QLD 4072, Australia; 24 Queensland Brain Institute, The University of Queensland, St Lucia QLD 4065, Australia; 25 National Centre for Register-based Research, Aarhus University, Aarhus V 8000, Denmark; 26 National Institute of Psychiatry Ramón de la Fuente Muñiz, Mexico City, Mexico; 27 UDIF-SM, Servicio Murciano de Salud. IMIB-Arrixaca. CIBERESP-Murcia, Región de Murcia, Spain; 28 Department of Mental Health, Graduate School of Medicine, The University of Tokyo, Tokyo, Japan; 29 Instituto Nacional de Salud, Lima, Peru; 30 Universidad Cayetano Heredia, Lima, Peru; 31 Colegio Mayor de Cundinamarca University, Faculty of Social Sciences, Bogota, Colombia; 32 Department of Psychological Medicine, University of Otago, Dunedin, Otago, New Zealand; 33 Departamento de Psiquiatría y Salud Mental, Facultad de Medicina, Universidad de Buenos Aires, Argentina; 34 Department of Psychiatry & Mental Health and South African Medical Council Research Unit on Risk and Resilience in Mental Disorders, University of Cape Town and Groote Schuur Hospital, Cape Town, Republic of South Africa; 35 Center for Excellence on Research in Mental Health, CES University, Medellin, Colombia; 36 Department of Social Medicine, Postgraduate Program in Public Health, Federal University of Espírito Santo, Vitoria, Brazil; 37 Department of Mental Health, National Center of Public Health and Analyses, Sofia, Bulgaria; 38 Department of Health Care Policy, Harvard Medical School, Boston, Massachusetts, USA

**Keywords:** Comorbidity, impairment, major depressive disorder, suicidal thoughts and behaviours

## Abstract

**Aims:**

Major depressive disorder (MDD) is characterised by a recurrent course and high comorbidity rates. A lifespan perspective may therefore provide important information regarding health outcomes. The aim of the present study is to examine mental disorders that preceded 12-month MDD diagnosis and the impact of these disorders on depression outcomes.

**Methods:**

Data came from 29 cross-sectional community epidemiological surveys of adults in 27 countries (*n* = 80 190). The Composite International Diagnostic Interview (CIDI) was used to assess 12-month MDD and lifetime DSM-IV disorders with onset prior to the respondent's age at interview. Disorders were grouped into depressive distress disorders, non-depressive distress disorders, fear disorders and externalising disorders. Depression outcomes included 12-month suicidality, days out of role and impairment in role functioning.

**Results:**

Among respondents with 12-month MDD, 94.9% (s.e. = 0.4) had at least one prior disorder (including previous MDD), and 64.6% (s.e. = 0.9) had at least one prior, non-MDD disorder. Previous non-depressive distress, fear and externalising disorders, but not depressive distress disorders, predicted higher impairment (OR = 1.4–1.6) and suicidality (OR = 1.5–2.5), after adjustment for sociodemographic variables. Further adjustment for MDD characteristics weakened, but did not eliminate, these associations. Associations were largely driven by current comorbidities, but both remitted and current externalising disorders predicted suicidality among respondents with 12-month MDD.

**Conclusions:**

These results illustrate the importance of careful psychiatric history taking regarding current anxiety disorders and lifetime externalising disorders in individuals with MDD.

## Introduction

Depressive disorders are ranked as the third leading cause of ‘years lived with disability’ worldwide (GBD 2017 Disease and Injury Incidence and Prevalence Collaborators, 2018). This is due in part to their high prevalence, with a lifetime prevalence of 10.6% and a 12-month prevalence of 4.5% for major depressive disorder (MDD) (Bromet *et al*., 2018). Additionally, MDD is often characterised by a recurrent course (Hardeveld *et al*., 2013), suggesting the importance of taking a lifespan perspective when studying MDD (Hardeveld *et al*., 2010; Bockting *et al*., 2015).

Comorbidity with other mental disorders is high in individuals with MDD (e.g. Grant, 1995; Kessler *et al*., 2003; Alonso and Lépine, 2007; Bromet *et al*., 2018). Since MDD has a rather late median age of onset (AOO) (Kessler and Bromet, 2013), i.e. around 38 years (Bromet *et al*., 2018), other mental disorders with earlier AOOs, such as anxiety disorders (Goodwin, 2002; Bittner *et al*., 2004; Beesdo *et al*., 2007; Mathew *et al*., 2011; Kessler *et al*., 2012; Gundel *et al*., 2018) and behavioural disorders (including oppositional defiant disorder (ODD), conduct disorder (CD) and attention deficit hyperactivity disorder (ADHD)) (Kessler *et al*., 2012; Gundel *et al*., 2018) often precede first-onset MDD. Assessing the clinical history of mental disorders in individuals with current MDD could provide valuable information regarding management and treatment strategies, especially since this history may be a prognostic marker of the further clinical course of MDD (Wittchen *et al*., 2003). Mental disorder comorbidity is predictive of depression outcomes, including impairment (Andrews *et al*., 2001; Alonso *et al*., 2004; Kessler *et al*., 2015) and suicidal thoughts and behaviours (Stein *et al*., 2001; Bolton *et al*., 2010; Kessler *et al*., 2015). However, to our knowledge, no study has comprehensively examined the associations between a wide range of mental disorders with an onset before 12-month MDD diagnosis with multiple depression outcomes.

The overall aim of the present study is to examine associations between 12-month MDD, previous mental disorders and MDD outcomes. First, we examine the strength of associations between 12-month MDD and preceding individual lifetime mental disorders in the cross-national World Mental Health (WMH) surveys. Second, we examine whether previous mental disorders, i.e. with an onset prior to the respondent's age at interview, are predictive of current depression outcomes including impairment and suicidality.

## Methods

### WMH surveys

Data came from 29 cross-sectional surveys administered between 2001/2002 and 2015 in low/middle-income countries (Brazil, Bulgaria, Colombia, Colombia (Medellin), China (Shenzhen), Iraq, Lebanon, Mexico, Nigeria, Peru, Romania, South Africa and Ukraine) and high-income countries (Argentina, Australia, Belgium, France, Germany, Israel, Italy, Japan, the Netherlands, New Zealand, Northern Ireland, Poland, Portugal, Spain, Spain (Murcia), USA). A total of 145 990 respondents participated. Adults were selected based on multistage clustered area probability sampling designs designed to generate samples that were representative of the household populations in each country. Face-to-face interviews were conducted in respondent homes. The details of sampling methods in the different countries are described in detail elsewhere (Heeringa *et al*., 2008; Pennell *et al*., 2008). The weighted average response rate across surveys was 69.5%. Informed consent was obtained according to protocols endorsed by local Institutional Review Boards. Further information on the surveys is provided in online Supplementary Table 1.

### MDD and other comorbid DSM-IV diagnoses

The WHO Composite International Diagnostic Interview (CIDI), a fully structured interview administered by trained lay interviewers, was used to assess DSM-IV mental disorders (Kessler and Üstün, 2004). Interviews were administered in two parts to diminish respondent burden. All respondents completed Part I, assessing MDD and other core mental disorders. Part II, assessing other disorders and disorder correlates, was administered to all respondents with any lifetime Part I diagnosis and a probability subsample of other respondents. Part II data were weighted to adjust for the undersampling of Part I non-cases. Analyses for this study were performed in the Part II sample (*N* = 80 190).

Since mental disorders are significantly correlated, which may be indicative of shared underlying vulnerability factors, a model-based approach of comorbidity of mental disorders was used (Krueger and Markon, 2006). Grouping of DSM-IV disorders was based on the underlying structure of disorders found previously in the WMH surveys (de Jonge *et al*., 2018). Given our focus on MDD, we separated two groups of distress disorders: depressive distress disorders (MDD and dysthymia (hierarchical)) and non-depressive distress disorders (generalised anxiety disorder (GAD) and post-traumatic stress disorder (PTSD)). Fear disorders included agoraphobia (non-hierarchical), panic disorder, social anxiety disorder, specific phobia and (childhood or adult) separation anxiety disorder. Externalising disorders included intermittent explosive disorder, ADHD, CD, ODD, alcohol abuse, alcohol dependence, drug abuse and drug dependence. The AOO of disorders was assessed using special recall probes that have been shown to yield more plausible distributions of AOO of disorders than conventional recall questions (Knäuper *et al*., 1999). Lifetime comorbid disorders were taken into account when they had an onset prior to the respondent's age at interview, regardless of whether or not they persisted into the past year.

### Outcomes

#### Role impairment

Role impairment due to MDD was assessed with a modified version of the Sheehan Disability Scale (SDS) (Leon *et al*., 1997). This scale assesses impairment in different domains, namely home management, ability to work, ability to form and maintain close relationships, and social life, during the month in the past year when MDD was most severe. Severe impairment was defined as a score ⩾7 on a response scale of 0–10 for each domain. Respondents were defined as having any severe impairment if they scored ⩾7 in at least one domain. Additionally, respondents were asked how many days in the past year they were unable to work or carry out their normal activities because of their depression, i.e. days out of role (Ormel *et al*., 2008).

#### Suicidal thoughts and behaviours

Respondents were asked whether they had ever seriously thought about suicide, and if so, whether they had ever made a suicide plan or attempted suicide. Respondents who reported these experiences were asked whether these experiences had also occurred in the past 12 months.

### Statistical analysis

The actuarial method was used to calculate the median AOO of MDD in respondents with 12-month MDD. We used logistic regression to examine the association between 12-month MDD and other mental disorders (controlling for country of origin). If a specific disorder was not assessed in a particular country, that country was not included in that analysis; however, if a country assessed at least one of the disorders in a disorder group (e.g. fear disorders), the country was included in the analysis of that disorder group, and non-assessed disorders were assumed not to be present.

We also used logistic regression to examine the association between previous mental disorder groups and depression outcomes (role impairment and suicidality). Linear regression was used for days out of role due to MDD in the past year, a continuous outcome. The models included all disorder groups simultaneously. We tested three models: model 1 only controlled for country of origin of the participant; model 2 additionally adjusted for age and sex; and model 3 additionally adjusted for educational status, marital status and employment status.

In post-hoc analyses, we added depression characteristics, namely AOO of MDD, lifetime history of chronic MDD (duration of longest episode >2 years) and severity of MDD in the worst month of the past year (according to the Quick Inventory of Depressive Symptomatology Self Report (QIDS-SR)) (Rush *et al*., 2000), to model 3. Furthermore, in a previous study, current, but not remitted anxiety disorders and dysthymia, predicted MDD episode duration (ten Have *et al*., 2017). As our main analyses include both remitted disorders and disorders that had onset prior to the current age but persisted into the past year, we distinguished remitted (i.e. no 12-month diagnosis) from current disorders in additional post-hoc analyses.

## Results

### Sample characteristics

A full description of the sample's characteristics stratified by 12-month MDD status is presented in online Supplementary Table 2. The number of respondents who met the criteria for 12-month MDD was 6684. The (weighted) prevalence of 12-month MDD was 4.9% (standard error (s.e.) = 0.1). Of the respondents with 12-month MDD, 66.8% were female. In this group, the median age of MDD onset was 38 years (interquartile range = 23–54) and 34.4% had a lifetime history of chronic MDD.

### History of mental disorders

Among respondents with 12-month MDD, 92.0% (s.e. = 0.5) had a prior history of MDD, and 94.9% (s.e. = 0.4) had a history of any previous mental disorder (including previous MDD). After excluding prior MDD episodes, this percentage dropped to 64.6% (s.e. = 0.9). Compared to respondents without 12-month MDD, prevalence rates of all other mental disorders with an onset prior to the respondent's age at interview were statistically significantly increased in respondents with 12-month MDD. The ORs ranged from 1.7 (alcohol abuse) to 20.2 (dysthymia) (see Table 1). ORs were largest for depressive distress disorders (including previous MDD) (OR = 161.1; 95% CI 140.0–185.4; *p* < 0.001), followed by non-depressive distress disorders (OR = 7.4; 95% CI 6.8–8.0; *p* < 0.001), fear disorders (OR = 4.8; 95% CI 4.5–5.2; *p* < 0.001) and externalising disorders (OR = 2.3; 95% CI 2.1–2.5; *p* < 0.001).

**Table 1.** Lifetime prevalence of previous disorders among respondents with 12-month MDD and respondents without 12-month MDD

Disorders	Respondents without 12-month MDD		Respondents with 12-month MDD		Estimates		
	%	s.e.	%	s.e.	OR	95% CI	*p*
MDD	6.9	0.1	92.0	0.5	164.7	143.1–189.5	<0.001
Dysthymia	0.6	0.0	12.0	0.6	20.2	17.5–23.4	<0.001
Any depressive distress disorder	7.2	0.1	92.1	0.5	161.1	140.0–185.4	<0.001
Generalised anxiety disorder	3.4	0.1	22.8	0.7	7.7	7.0–8.5	<0.001
Post-traumatic stress disorder	3.1	0.1	15.7	0.6	5.4	4.8–6.0	<0.001
Any non-depressive distress disorder	5.6	0.1	32.0	0.8	7.4	6.8–8.0	<0.001
Agoraphobia	1.5	0.1	6.9	0.4	4.6	3.9–5.3	<0.001
Panic disorder	1.5	0.1	8.4	0.5	5.4	4.7–6.1	<0.001
Separation anxiety disorder	4.1	0.1	17.1	0.8	4.2	3.7–4.8	<0.001
Social anxiety disorder	4.0	0.1	17.6	0.7	4.6	4.2–5.1	<0.001
Specific phobia	7.4	0.1	23.7	0.8	3.4	3.1–3.8	<0.001
Any fear disorder	11.2	0.2	38.2	0.8	4.8	4.5–5.2	<0.001
Intermittent explosive disorder	2.9	0.1	10.9	0.6	3.2	2.8–3.8	<0.001
Attention deficit hyperactivity disorder	1.7	0.1	5.9	0.5	2.8	2.2–3.6	<0.001
Conduct disorder	1.9	0.1	5.0	0.5	2.0	1.5–2.5	<0.001
Oppositional defiant disorder	2.3	0.1	6.3	0.6	2.4	2.0–2.9	<0.001
Alcohol abuse	9.0	0.1	15.7	0.6	1.7	1.6–1.9	<0.001
Alcohol dependence	2.3	0.1	7.3	0.4	3.0	2.6–3.5	<0.001
Drug abuse	3.1	0.1	8.0	0.5	2.5	2.2–2.9	<0.001
Drug dependence	1.0	0.1	4.0	0.3	3.4	2.7–4.1	<0.001
Any externalising disorder	12.3	0.2	26.0	0.7	2.3	2.1–2.5	<0.001

MDD, major depressive disorder; s.e., standard error; OR, odds ratio; CI, confidence interval.

Analyses controlled for country.

### Role impairment

Overall impairment rates in respondents with 12-month MDD were 58.6% (s.e. = 0.9) for any severe impairment, 35.2% (s.e. = 0.9) for severe impairment in home management, 35.6% (s.e. = 0.9) for severe impairment in ability to work, 33.7% (s.e. = 0.9) for severe impairment in the ability to form and maintain close relationships, and 40.2% (s.e. = 0.9) for severe impairment in social life.

Among respondents with 12-month MDD, those with a previous depressive distress disorder were less likely to be severely impaired due to MDD in home management (OR = 0.6; 95% CI 0.4–0.8; *p* = 0.001), ability to work (OR = 0.6; 95% CI 0.4–0.8; *p* < 0.001) and social life (OR = 0.6; 95% CI 0.4–0.8; *p* < 0.001) compared to respondents without a previous mental disorder (see model 1, Table 2). However, the association between history of depressive distress disorders and ability to form and maintain close relationships was not statistically significant (OR = 0.7; 95% CI 0.5–1.0; *p* = 0.061). Respondents with a previous non-depressive distress disorder (GAD or PTSD) or fear disorder had significantly increased odds (ORs ranging from 1.3 to 1.7) of impairment due to MDD in all roles, compared to respondents without previous mental disorders. Respondents with a previous externalising disorder also had an increased risk of impairment in ability to work, social life and relationships (ORs = 1.2–1.3), but not of impairment in home management (OR = 1.0; 95% CI 0.8–1.2; *p* = 0.96). Adjustment for sex and age in model 2, and additionally for education, marital status and employment in model 3, did not change these findings (see Table 2).

**Table 2.** Association between previous disorders and severe role impairment

	Home			Work			Relationship			Social			Any		
	OR	95% CI	*p*	OR	95% CI	*p*	OR	95% CI	*p*	OR	95% CI	*p*	OR	95% CI	*p*
Model 1															
No previous disorders (ref)	–			–			–			–			–		
Depressive distress disorders	0.6	0.4–0.8	0.001	0.6	0.4–0.8	<0.001	0.7	0.5–1.0	0.061	0.6	0.4–0.8	<0.001	0.7	0.5–0.9	0.004
Non-depressive distress disorders	1.7	1.4–2.0	<0.001	1.6	1.3–1.9	<0.001	1.7	1.4–2.0	<0.001	1.7	1.4–2.0	<0.001	1.6	1.4–1.9	<0.001
Fear disorders	1.3	1.1–1.6	<0.001	1.3	1.1–1.6	0.003	1.4	1.2–1.7	<0.001	1.5	1.2–1.7	<0.001	1.5	1.3–1.8	<0.001
Externalising disorders	1.0	0.8–1.2	0.96	1.2	1.0–1.5	0.025	1.3	1.1–1.5	0.004	1.3	1.1–1.5	0.006	1.3	1.1–1.6	0.001
Model 2															
No previous disorders (ref)	–			–			–			–			–		
Depressive distress disorders	0.5	0.4–0.8	<0.001	0.5	0.4–0.8	<0.001	0.7	0.5–1.0	0.073	0.6	0.4–0.8	<0.001	0.6	0.5–0.9	0.004
Non-depressive distress disorders	1.6	1.3–1.9	<0.001	1.5	1.3–1.8	<0.001	1.7	1.4–2.0	<0.001	1.7	1.4–2.0	<0.001	1.6	1.3–1.9	<0.001
Fear disorders	1.3	1.1–1.6	0.002	1.3	1.1–1.6	0.002	1.4	1.2–1.7	<0.001	1.5	1.2–1.7	<0.001	1.5	1.3–1.8	<0.001
Externalising disorders	1.2	1.0–1.5	0.11	1.3	1.1–1.6	0.010	1.3	1.0–1.5	0.017	1.3	1.1–1.6	0.005	1.4	1.1–1.7	0.002
Model 3															
No previous disorders (ref)	–			–			–			–			–		
Depressive distress disorders	0.5	0.4–0.7	<0.001	0.5	0.4–0.7	<0.001	0.7	0.5–1.0	0.051	0.6	0.4–0.8	<0.001	0.6	0.5–0.8	0.002
Non-depressive distress disorders	1.6	1.3–1.9	<0.001	1.5	1.2–1.8	<0.001	1.7	1.4–2.0	<0.001	1.6	1.4–1.9	<0.001	1.6	1.3–1.9	<0.001
Fear disorders	1.3	1.1–1.5	0.004	1.3	1.1–1.6	0.007	1.4	1.2–1.7	<0.001	1.5	1.2–1.7	<0.001	1.5	1.2–1.7	<0.001
Externalising disorders	1.1	0.9–1.4	0.21	1.3	1.0–1.6	0.016	1.2	1.0–1.5	0.032	1.3	1.1–1.6	0.010	1.4	1.1–1.7	0.003

MDD, major depressive disorder; OR, odds ratio; CI, confidence interval.

Association between severe role impairment and previous depressive distress, non-depressive distress, fear or externalising disorders, among respondents with 12-month MDD.

Depressive distress disorders: major depressive disorder and dysthymia; non-depressive distress disorders: generalised anxiety disorder and post-traumatic stress disorder; fear disorders: agoraphobia, panic disorder, separation anxiety disorder, social anxiety disorder, specific phobia; externalising disorders: intermittent explosive disorder, attention deficit hyperactivity disorder, conduct disorder, oppositional defiant disorder, alcohol abuse, alcohol dependence, drug abuse and drug dependence.

Model 1: adjusted for country.

Model 2: adjusted for country, age, sex.

Model 3: adjusted for country, age, sex, education, marital status and employment status.

### Days out of role

The mean number of days out of role in the past year due to MDD for participants with 12-month MDD was 36.4 (s.e. = 1.8). Compared to respondents with 12-month MDD without previous mental disorders, those with previous depressive distress disorders (*B* = 10.9; 95% CI 1.1–20.8; *p* = 0.030), non-depressive distress disorders (*B* = 16.6; 95% CI 8.4–24.8; *p* < 0.001) or fear disorders (*B* = 13.8; 95% CI 6.4–21.3; *p* < 0.001) had higher numbers of days out of role (online Supplementary Table 3). The number of days out of role was not significantly higher for respondents with previous externalising disorders (*B* = 5.1; 95% CI −2.5 to 12.6; *p* = 0.19). After adjustment for sex and age, the association for previous depressive distress disorders lost statistical significance (*B* = 9.3; 95% CI −0.3 to 18.8; *p* = 0.057). However, previous non-depressive distress disorders (*B* = 13.2; 95% CI 4.9–21.4; *p* = 0.002) and previous fear disorders (*B* = 14.1; 95% CI 6.8–21.4; *p* < 0.001) remained significant predictors of days out of role even in the fully adjusted model.

### Suicidal thoughts and behaviours

Of all respondents with 12-month MDD, 13.2% (s.e. = 0.5) reported suicidal ideation in the past 12 months and 4.4% (s.e. = 0.3) and 2.6% (s.e. = 0.2), respectively, reported suicide plans and attempts over this period. Previous depressive distress disorders were not significantly associated with 12-month suicidal ideation, plan or attempt in any model (ORs = 0.9–1.3) (Table 3). Previous non-depressive distress disorders were associated with suicidal ideation, plan and attempts in respondents with 12-month MDD, but not all associations were statistically significant in each model (ORs = 1.3–1.7) (see Table 3). Previous fear disorders (ORs = 1.5–1.7) and previous externalising disorders (ORs = 1.9–2.9) were consistently associated with suicidal ideation, plan and attempt in all models.

**Table 3.** Association between previous disorders and suicidality

	Ideation			Plan			Attempt		
	OR	95% CI	*p*	OR	95% CI	*p*	OR	95% CI	*p*
Model 1									
No previous disorders (ref)	–			–			–		
Depressive distress disorders	0.9	0.6–1.3	0.43	1.2	0.7–2.2	0.52	1.0	0.5–1.9	0.92
Non-depressive distress disorders	1.4	1.2–1.7	<0.001	1.3	0.9–1.8	0.13	1.4	1.0–2.1	0.081
Fear disorders	1.7	1.4–2.0	<0.001	1.7	1.2–2.3	0.001	1.6	1.1–2.4	0.023
Externalising disorders	2.1	1.7–2.6	<0.001	2.9	2.2–3.8	<0.001	2.3	1.6–3.4	<0.001
Model 2									
No previous disorders (ref)	–			–			–		
Depressive distress disorders	0.9	0.6–1.3	0.55	1.3	0.7–2.3	0.43	1.1	0.5–2.1	0.88
Non-depressive distress disorders	1.5	1.3–1.9	<0.001	1.4	1.0–2.0	0.032	1.7	1.1–2.5	0.010
Fear disorders	1.6	1.4–2.0	<0.001	1.7	1.2–2.3	0.002	1.5	1.0–2.3	0.043
Externalising disorders	1.9	1.6–2.3	<0.001	2.5	1.9–3.3	<0.001	2.0	1.3–3.0	0.002
Model 3									
No previous disorders (ref)	–			–			–		
Depressive distress disorders	0.9	0.6–1.3	0.43	1.2	0.7–2.3	0.49	1.0	0.5–2.0	0.96
Non-depressive distress disorders	1.5	1.2–1.8	<0.001	1.3	1.0–1.8	0.090	1.6	1.1–2.4	0.021
Fear disorders	1.6	1.3–2.0	<0.001	1.6	1.2–2.1	0.003	1.6	1.1–2.5	0.018
Externalising disorders	1.9	1.5–2.3	<0.001	2.5	1.9–3.3	<0.001	2.0	1.3–3.0	<0.001

MDD, major depressive disorder; OR, odds ratio, CI, confidence interval.

Association between suicidality and previous depressive distress, non-depressive distress, fear or externalising disorders, among respondents with 12-month MDD.

Depressive distress disorders: major depressive disorder and dysthymia; non-depressive distress disorders: generalised anxiety disorder and post-traumatic stress disorder; fear disorders: agoraphobia, panic disorder, separation anxiety disorder, social anxiety disorder, specific phobia; externalising disorders: intermittent explosive disorder, attention deficit hyperactivity disorder, conduct disorder, oppositional defiant disorder, alcohol abuse, alcohol dependence, drug abuse and drug dependence.

Model 1: adjusted for country.

Model 2: adjusted for country, age, sex.

Model 3: adjusted for country, age, sex, education, marital status and employment status.

### Post-hoc analyses including MDD characteristics

When depression characteristics were added to the models, a higher age of MDD onset and 12-month MDD severity were associated with role impairment due to MDD (online Supplementary Table 4). In this model, previous fear disorders were no longer associated with role impairment in any of the roles, except social life (OR = 1.2; 95% CI 1.0–1.5; *p* = 0.029). Associations between previous non-depressive distress disorders and previous externalising disorders with role impairment remained statistically significant (ORs = 1.1–1.4), with the exception of the association between previous externalising disorders and work impairment (OR = 1.1; 95% CI 0.9–1.4; *p* = 0.30).

Only previous fear disorders were associated with a higher number of days out of role compared to respondents with 12-month MDD without previous mental disorders (*B* = 7.2; 95% CI 0.0–14.4; *p* = 0.049) after additional adjustment for depression characteristics. Lifetime MDD chronicity and 12-month MDD severity were strongly related to days out of role in this model (online Supplementary Table 5).

Additionally, previous non-depressive distress disorders were no longer statistically significantly related to suicidal thoughts and behaviours. However, previous fear and externalising disorders (ORs = 1.4–2.1), as well as MDD severity, were associated with suicidal ideation, plans and attempts (online Supplementary Table 6).

### Post-hoc analyses on remitted disorders

After separating previous disorders into those still present in the past 12 months and those that had remitted, 52.3% (s.e. = 0.9) of participants with 12-month MDD had any current comorbidity, while 12.3% (s.e. = 0.5) only had remitted comorbidity. Previous mental disorders were only associated with role impairment when they were also present in the past 12 months, with the exception of remitted externalising disorders and social impairment (OR = 1.3; 95% CI 1.0–1.6; *p* = 0.027) (Table 4). Similarly, days out of role due to MDD and suicidal thoughts and behaviours were related to current, but not remitted, fear disorders (online Supplementary Table 7 and Table 5). Current externalising disorders were associated with suicidal ideation (OR = 2.0; 95% CI 1.5–2.5; *p* < 0.001) and plans (OR = 2.6; 95% CI 1.8–3.8, *p* < 0.001), but not with suicide attempts (OR = 1.6; 95% CI 0.9–2.7; *p* = 0.10). Remitted externalising disorders, however, were only significantly associated with suicide attempts (OR = 1.7; 95% CI 1.0–3.0; *p* = 0.0498) and not with suicidal ideation (OR = 1.3; 95% CI 1.0–1.8; *p* = 0.06) or plans (OR = 1.5; 95% CI 1.0–2.4; *p* = 0.07) (Table 5).

**Table 4.** Association between previous disorders, divided into remitted or current comorbid disorders, and severe role impairment

	Home			Work			Relationship			Social			Any impairment		
	OR	95% CI	*p*	OR	95% CI	*p*	OR	95% CI	*p*	OR	95% CI	*p*	OR	95% CI	*p*
Disorders															
No previous disorders (ref)	–			–			–			–			–		
Depressive distress disorders	0.4	0.3–0.6	<0.001	0.5	0.4–0.7	<0.001	0.7	0.5–1.0	0.05	0.6	0.4–0.8	0.001	0.6	0.4–0.8	0.002
Non-depressive distress disorders, remitted	1.0	0.7–1.4	0.97	1.2	0.8–1.6	0.36	1.2	0.8–1.6	0.35	1.0	0.8–1.4	0.79	1.0	0.8–1.4	0.78
Non-depressive distress disorders, current	1.3	1.1–1.7	0.005	1.3	1.0–1.6	0.023	1.5	1.2–1.8	<0.001	1.4	1.2–1.8	<0.001	1.4	1.1–1.8	0.002
Fear disorders, remitted	0.9	0.7–1.2	0.36	0.8	0.6–1.1	0.26	0.9	0.6–1.2	0.36	1.1	0.8–1.5	0.49	1.0	0.8–1.4	0.83
Fear disorders, current	0.9	0.8–1.2	0.61	1.0	0.8–1.2	0.97	1.2	1.0–1.5	0.039	1.3	1.0–1.6	0.020	1.2	1.0–1.5	0.08
Externalising disorders, remitted	1.0	0.8–1.4	0.78	1.1	0.8–1.5	0.51	1.2	0.9–1.5	0.16	1.3	1.0–1.6	0.027	1.3	1.0–1.7	0.07
Externalising disorders, current	1.2	0.9–1.7	0.18	1.1	0.9–1.5	0.35	1.3	0.9–1.7	0.11	1.5	1.1–2.1	0.006	1.7	1.2–2.3	<0.001

MDD, major depressive disorder; OR, odds ratio; CI, confidence interval.

Association between severe role impairment and previous depressive distress, non-depressive distress, fear or externalising disorders, among respondents with 12-month MDD. Previous disorders are divided into remitted and current disorders.

Depressive distress disorders: major depressive disorder and dysthymia; non-depressive distress disorders: generalised anxiety disorder and post-traumatic stress disorder; fear disorders: agoraphobia, panic disorder, separation anxiety disorder, social anxiety disorder, specific phobia; externalising disorders: intermittent explosive disorder, attention deficit hyperactivity disorder, conduct disorder, oppositional defiant disorder, alcohol abuse, alcohol dependence, drug abuse and drug dependence.

Analyses are adjusted for country, age, sex, education, marital status, employment status and MDD characteristics (age of onset, chronicity, severity).

**Table 5.** Association between previous disorders, divided into remitted or current comorbid disorders, and suicidality

	Ideation			Plan			Attempt		
	OR	95% CI	*p*	OR	95% CI	*p*	OR	95% CI	*p*
No previous disorder (ref)	–			–			–		
Depressive distress disorders	0.7	0.4–1.2	0.23	1.0	0.5–2.0	0.99	1.6	0.7–3.9	0.29
Non-depressive distress disorders (remitted)	0.7	0.5–1.1	0.10	0.5	0.2–0.9	0.029	0.9	0.4–2.0	0.86
Non-depressive distress disorders (current)	1.2	0.9–1.6	0.12	1.2	0.8–1.7	0.48	1.1	0.7–1.8	0.63
Fear disorders (remitted)	0.7	0.5–1.1	0.16	1.1	0.6–1.9	0.81	1.6	0.9–3.1	0.13
Fear disorders (current)	1.5	1.2–1.9	<0.001	1.5	1.0–2.1	0.029	1.7	1.1–2.7	0.024
Externalising disorders (remitted)	1.3	1.0–1.8	0.06	1.5	1.0–2.4	0.07	1.7	1.0–3.0	0.050
Externalising disorders (current)	2.0	1.5–2.5	<0.001	2.6	1.8–3.8	<0.001	1.6	0.9–2.7	0.10

MDD, major depressive disorder; OR, odds ratio; CI, confidence interval.

Association between suicidality and previous depressive distress, non-depressive distress, fear or externalising disorders, among respondents with 12-month MDD. Previous disorders are divided into remitted and current disorders.

Depressive distress disorder: major depressive disorder and dysthymia; non-depressive distress disorder: generalised anxiety disorder and post-traumatic stress disorder; fear disorder: agoraphobia, panic disorder, separation anxiety disorder, social anxiety disorder, specific phobia; externalising disorders: intermittent explosive disorder, attention deficit hyperactivity disorder, conduct disorder, oppositional defiant disorder, alcohol abuse, alcohol dependence, drug abuse and drug dependence.

Analyses adjusted for country, age, sex, education, marital status, employment status and MDD characteristics (age on onset, chronicity and severity).

## Discussion

To our knowledge, this is the first epidemiological study that comprehensively examined the history of mental disorders in individuals with current MDD and their impact on depression outcomes. The results of this study show the importance of applying a lifespan perspective when studying MDD. We found that 95% of respondents with 12-month MDD had a previous mental disorder. Many had suffered from previous MDD episodes, confirming the recurrent and/or chronic nature of MDD and demonstrating that first-onset cases are rare among people with a 12-month MDD episode. However, even after excluding previous MDD, 65% of respondents reported previous mental disorders. Compared to respondents without MDD, those with MDD had higher lifetime prevalence rates of all other individual mental disorders.

### Previous disorders and depression outcomes

Importantly, previous mental disorders in respondents with MDD were associated with impairment and suicidality. Previous non-depressive distress disorders and fear disorders were most strongly associated with impairment. These results are consistent with other studies that reported high impairment rates in individuals with MDD and comorbid anxiety disorders (Wittchen *et al*., 2003; Van Der Werff *et al*., 2010; Kessler *et al*., 2015) and reinforce the clinical importance of anxiety symptoms in MDD (Gaspersz *et al*., 2018). Further, previous non-depressive distress, fear and externalising disorders were related to past-year suicidal ideation, plans and attempts in respondents with 12-month MDD. The strongest associations were found for previous externalising disorders, consistent with other epidemiological studies that found associations between externalising psychopathology and suicide attempts independent from internalising psychopathology (Verona *et al*., 2004; Hills *et al*., 2005, 2009).

Surprisingly, previous depressive distress disorders were associated with less frequent severe role impairment. However, respondents with previous depressive distress disorders did have a higher number of days out of role compared to respondents with first-onset MDD, although this association did not remain statistically significant after adjustment for covariates. Individuals with recurrent or chronic MDD may be less likely to report severe impairment in different domains as a result of habituation or adaptation to depressive episodes (Kruijshaar *et al*., 2003). For example, it is possible that recurrent or chronic MDD has led to the individual having reduced activity in some roles (e.g. being under-employed); an ability to function adequately in a reduced role may or may not be interpreted as impairment. However, individuals with recurrent or chronic MDD may also have reduced insight regarding impairment. The finding that people with previous depression do report a higher number of days out of role, a more objective measure of impairment, argues that reduced insight may play some role, yet this is a post-hoc speculation. Previous research has found conflicting results, with some other studies reporting that recurrence of MDD was related to increased functional impairment and work disability (Merikangas *et al*., 1994; Rytsälä *et al*., 2005), while others also found lower reported impairment among those with recurrent MDD (Kruijshaar *et al*., 2003; Van Der Werff *et al*., 2010), although this latter finding lost statistical significance after adjustment for depression characteristics, including symptom severity (Van Der Werff *et al*., 2010). Interestingly, we found that a history of depressive distress disorders remained a protective factor for role impairment even after adjustment for MDD characteristics, including severity.

Somewhat surprisingly, a history of depressive disorder was not associated with 12-month suicidal ideation, plan or attempt. This suggests that people with a longer history of depression are not currently at higher risk of suicidal thoughts and behaviours than people with first onset of depression. Nevertheless, it is likely that people with a longer history of depression do have a higher lifetime risk of suicidality as risk increases over the years since persons with (a history of) MDD remain vulnerable for suicidal ideation over time (de Beurs *et al*., 2019).

### Lifespan perspective to MDD and other mental disorders

Applying a lifespan perspective to MDD appears to be of importance, since individuals with MDD are highly likely to have suffered from previous MDD and other mental disorders and these disorders predict current, and potentially also future, depression outcomes. Other epidemiological studies have also shown that comorbidity is associated with increased depressive episode duration (ten Have *et al*., 2017), lower likelihood of MDD remission (Kelly and Mezuk, 2017) and chronicity of MDD (Satyanarayana *et al*., 2009; Murphy and Byrne, 2012; ten Have *et al*., 2018). On the other hand, research findings have not been consistent. For example, Hoertel *et al*. (2015) concluded that patient accounts of previous mental disorders would probably not improve the prediction of future suicidality, because current comorbid mental disorders explained the associations of remitted mental disorders with suicide attempts. Our post-hoc analyses also showed that most of the associations between comorbid mental disorders and outcomes were explained by current, rather than remitted, disorders, but our results contrast with Hoertel *et al*.'s since we found that both current and remitted externalising disorders were associated with 12-month suicidal thoughts and behaviours. Another study also suggested residual effects of (comorbid) psychopathology, finding that individuals with previous internalising and externalising disorders still reported higher functional impairment even 12 months after remission of the disorder (Bijl and Ravelli, 2000).

Potential benefits of taking a lifespan perspective when examining mental disorders are probably not restricted to MDD. Future studies could examine the role of previous disorders in onset and associated disability of other mental disorders with a relatively late median AOO such as panic disorder, or instead, focus on early-onset disorders as causes or markers of future psychopathology. For example, in another report of the WMH surveys, specific phobia in childhood was predictive of other internalising disorders as well as impairment throughout the life course (de Vries *et al*., 2019).

### Comparison with clinical studies

The results of the current study are in line with clinical studies. Among outpatients with a primary diagnosis of current MDD, 91% had another lifetime mental disorder diagnosis (including previous MDD) (Brown *et al*., 2001). Another study in outpatients with MDD also showed high prevalence rates of lifetime and current comorbidity (Zimmerman *et al*., 2002; Zimmerman *et al*., 2008). However, these findings do raise the question of whether these high comorbidity rates represent an artefact of current diagnostic systems, for example, as a result of the increasing number of diagnoses in the DSM (Maj, 2005*a*), while different diagnoses may actually result from a single clinical entity (Maj, 2005*b*). Nevertheless, detecting the presence of comorbid psychiatric symptoms may be very relevant in clinical management (Mai, 2005*a*). Indeed, the importance of taking comorbidity into account was exemplified by Zimmerman *et al*. (2002); their study showed that the majority of patients with primary MDD and comorbidity wanted treatment to also focus on these comorbid current disorders, partially remitted disorders or symptoms not meeting full diagnostic criteria (Zimmerman *et al*., 2002). Unfortunately, underrecognition of comorbidity is likely to be common in routine clinical care when unstructured clinical evaluations are used and time is limited (Zimmerman *et al*., 2008).

The importance of a lifetime approach to MDD in clinical care has been advocated before, for example, by Bockting *et al*. who argued that knowledge on risk factors of MDD recurrence, such as number of previous MDD episodes, may guide treatment choices (Bockting *et al*., 2015). The current study suggests the importance of additionally taking other previous mental disorders into account to further enhance personalised treatment, especially since most associations between previous lifetime comorbid disorders and current MDD outcomes remained statistically significant even after adjustment for MDD characteristics.

### Strengths and limitations

An important strength of the current study is the large, cross-national sample, providing the opportunity to examine the associations between MDD and histories of individual mental disorders. Another strength is the comprehensive assessment of multiple depression outcomes and post-hoc analyses that adjusted for MDD characteristics and separated remitted from current comorbid mental disorders. Yet this study also has several limitations. First, disorders were retrospectively assessed, so recall bias may be present, particularly with regard to retrospectively-reported AOO (Johnson *et al*., 1997). Second, although we distinguished first-onset from non-first-onset MDD episodes, we did not distinguish recurrent from persistent or chronic 12-month episodes. However, post-hoc analyses were adjusted for lifetime presence of chronic MDD. We also did not consider the duration or severity of comorbid disorders, although it is likely that more long-lasting and more severe comorbid disorders are more strongly associated with MDD outcomes. Finally, we did not consider the potential influence of prior treatment for either MDD or comorbid disorders.

## Conclusions

In this general population sample, almost all individuals with 12-month MDD had suffered from previous mental disorders. Among these individuals, previous non-depressive distress and fear disorders were particularly strongly associated with greater role impairment, confirming the clinical importance of anxiety in MDD. Previous externalising disorders were most strongly associated with suicidal ideation, plans and attempts. The risks of adverse depression outcomes associated with previous mental disorders were reduced in strength, but did not disappear, after adjustment for MDD characteristics. Risks were mostly conveyed through current, rather than remitted, previous mental disorders, except for the risk of impairment in social life and suicide attempts associated with remitted externalising disorders. These results illustrate the importance of careful psychiatric history taking regarding current anxiety disorders and lifetime externalising disorders in individuals with MDD.

## Data Availability

Access to the cross-national World Mental Health (WMH) data is governed by the organisations funding and responsible for survey data collection in each country. These organisations made data available to the WMH consortium through restricted data sharing agreements that do not allow us to release the data to third parties. The exception is that the US data are available for secondary analysis via the Inter-University Consortium for Political and Social Research (ICPSR), http://www.icpsr.umich.edu/icpsrweb/ICPSR/series/00527.

## Supplementary material

For supplementary material accompanying this paper visit http://dx.doi.org/10.1017/S2045796021000573. click here to view supplementary material
