# Supplementary material for: Previous disorders and depression outcomes in individuals with 12-month major depressive disorder in the World Mental Health surveys
Source: Epidemiol Psychiatr Sci. 2021 Nov 11;30:e70. doi: 10.1017/S2045796021000573 (PMC8611932; doi:10.1017/S2045796021000573)
Supplement: Supplementary file 1 [file S2045796021000573sup001.docx]

| **Supplemental table 1. WMH sample characteristics by World Bank income categories*^a^*.** | | | | | | | | |
| --- | --- | --- | --- | --- | --- | --- | --- | --- |
|  |  |  |  |  | **Sample size** | | |  |
| **Country by income category** | **Survey*^b^*** | **Sample characteristics*^c^*** | **Field dates** | **Age range** | **Part I** | **Part II** | **Part II and age ≤ 44*^d^*** | **Response rate*^e^*** |
| **I. Low and middle income countries** | | |  |  |  |  |  |  |
| Brazil - São Paulo | São Paulo Megacity | São Paulo metropolitan area. | 2005-8 | 18-93 | 5,037 | 2,942 | -- | 81.3 |
| Bulgaria | NSHS | Nationally representative. | 2002-6 | 18-98 | 5,318 | 2,233 | 741 | 72.0 |
| Colombia | NSMH | All urban areas of the country (approximately 73% of the total national population). | 2003 | 18-65 | 4,426 | 2,381 | 1,731 | 87.7 |
| Colombia – Medellin^f^ | MMHHS | Medellin metropolitan area | 2011-12 | 19-65 | 3,261 | 1,673 | -- | 97.2 |
| Iraq | IMHS | Nationally representative. | 2006-7 | 18-96 | 4,332 | 4,332 | -- | 95.2 |
| Lebanon | LEBANON | Nationally representative. | 2002-3 | 18-94 | 2,857 | 1,031 | 595 | 70.0 |
| Mexico | M-NCS | All urban areas of the country (approximately 75% of the total national population). | 2001-2 | 18-65 | 5,782 | 2,362 | 1,736 | 76.6 |
| Nigeria | NSMHW | 21 of the 36 states in the country, representing 57% of the national population. The surveys were conducted in Yoruba, Igbo, Hausa and Efik languages. | 2002-4 | 18-100 | 6,752 | 2,143 | 1,203 | 79.3 |
| Peru | EMSMP | Five urban areas of the country (approximately 38% of the total national population). | 2004-5 | 18-65 | 3,930 | 1,801 | 1,287 | 90.2 |
| PRC^g^  - Shenzhen^h^ | Shenzhen | Shenzhen metropolitan area. Included temporary residents as well as household residents. | 2005-7 | 18-88 | 7,132 | 2,475 | -- | 80.0 |
| Romania | RMHS | Nationally representative. | 2005-6 | 18-96 | 2,357 | 2,357 | -- | 70.9 |
| South Africa^h^ | SASH | Nationally representative. | 2002-4 | 18-92 | 4,315 | 4,315 | -- | 87.1 |
| Ukraine | CMDPSD | Nationally representative. | 2002 | 18-91 | 4,725 | 1,720 | 541 | 78.3 |
| **TOTAL** |  |  |  |  | (60,224) | (31,765) | (7,834) | 81.1 |
| **II. High-income countries** | | |  |  |  |  |  |  |
| Argentina | AMHES | Eight largest urban areas of the country (approximately 50% of the total national population) | 2015 | 18-98 | 3,927 | 2,116 | -- | 77.3 |
| Australia^h^ | NSMHWB | Nationally representative. | 2007 | 18-85 | 8,463 | 8,463 | -- | 60.0 |
| Belgium | ESEMeD | Nationally representative. The sample was selected from a national register of Belgium residents. | 2001-2 | 18-95 | 2,419 | 1,043 | 486 | 50.6 |
| France | ESEMeD | Nationally representative. The sample was selected from a national list of households with listed telephone numbers. | 2001-2 | 18-97 | 2,894 | 1,436 | 727 | 45.9 |
| Germany | ESEMeD | Nationally representative. | 2002-3 | 19-95 | 3,555 | 1,323 | 621 | 57.8 |
| Israel | NHS | Nationally representative. | 2003-4 | 21-98 | 4,859 | 4,859 | -- | 72.6 |
| Italy | ESEMeD | Nationally representative. The sample was selected from municipality resident registries. | 2001-2 | 18-100 | 4,712 | 1,779 | 853 | 71.3 |
| Japan | WMHJ 2002-2006 | Eleven metropolitan areas. | 2002-6 | 20-98 | 4,129 | 1,682 | -- | 55.1 |
| Netherlands | ESEMeD | Nationally representative. The sample was selected from municipal postal registries. | 2002-3 | 18-95 | 2,372 | 1,094 | 516 | 56.4 |
| New Zealand^h^ | NZMHS | Nationally representative. | 2004-5 | 18-98 | 12,790 | 7,312 | -- | 73.3 |
| N. Ireland | NISHS | Nationally representative. | 2005-8 | 18-97 | 4,340 | 1,986 | -- | 68.4 |
| Poland | EZOP | Nationally representative | 2010-11 | 18-65 | 10,081 | 4,000 | 2,276 | 50.4 |
| Portugal | NMHS | Nationally representative. | 2008-9 | 18-81 | 3,849 | 2,060 | 1,070 | 57.3 |
| Spain | ESEMeD | Nationally representative. | 2001-2 | 18-98 | 5,473 | 2,121 | 960 | 78.6 |
| Spain-Murcia | PEGASUS- Murcia | Murcia region. Regionally representative. | 2010-12 | 18-96 | 2,621 | 1,459 | -- | 67.4 |
| United States | NCS-R | Nationally representative. | 2001-3 | 18-99 | 9,282 | 5,692 | 3,197 | 70.9 |
| **TOTAL** |  |  |  |  | (85,766) | (48,425) | (10,706) | 63.1 |
| **III. TOTAL** |  |  |  |  | (145,990) | (80,190) | (18,540) | 69.5 |
|  | | | | | | | | |

*^a^* The World Bank (2012) Data. Accessed May 12, 2012 at: <http://data.worldbank.org/country>. Some of the WMH countries have moved into new income categories since the surveys were conducted. The income groupings above reflect the status of each country at the time of data collection. The current income category of each country is available at the preceding URL.

*^b^* NSHS (Bulgaria National Survey of Health and Stress); NSMH (The Colombian National Study of Mental Health); MMHHS (Medellín Mental Health Household Study); IMHS (Iraq Mental Health Survey); LEBANON (Lebanese Evaluation of the Burden of Ailments and Needs of the Nation); M-NCS (The Mexico National Comorbidity Survey); NSMHW (The Nigerian Survey of Mental Health and Wellbeing); EMSMP (La Encuesta Mundial de Salud Mental en el Peru); RMHS (Romania Mental Health Survey); SASH (South Africa Health Survey); CMDPSD (Comorbid Mental Disorders during Periods of Social Disruption); AMHES (Argentina Mental Health Epidemiologic Survey); NSMHWB (National Survey of Mental Health and Wellbeing); ESEMeD (The European Study Of The Epidemiology Of Mental Disorders); WMHJ2002-2006 (World Mental Health Japan Survey); NZMHS (New Zealand Mental Health Survey); NISHS (Northern Ireland Study of Health and Stress); EZOP (Epidemiology of Mental Disorders and Access to Care Survey); NMHS (Portugal National Mental Health Survey); PEGASUS-Murcia (Psychiatric Enquiry to General Population in Southeast Spain-Murcia); NCS-R (The US National Comorbidity Survey Replication).

*^c^* Most WMH surveys are based on stratified multistage clustered area probability household samples in which samples of areas equivalent to counties or municipalities in the US were selected in the first stage followed by one or more subsequent stages of geographic sampling (e.g., towns within counties, blocks within towns, households within blocks) to arrive at a sample of households, in each of which a listing of household members was created and one or two people were selected from this listing to be interviewed. No substitution was allowed when the originally sampled household resident could not be interviewed. These household samples were selected from Census area data in all countries other than France (where telephone directories were used to select households) and the Netherlands (where postal registries were used to select households). Several WMH surveys (Belgium, Germany, Italy, Poland, Spain-Murcia) used municipal, country resident or universal health-care registries to select respondents without listing households. The Japanese sample is the only totally un-clustered sample, with households randomly selected in each of the 11 metropolitan areas and one random respondent selected in each sample household. 19 of the 29 surveys are based on nationally representative household samples.

*^d^* Argentina, Australia, Brazil, Colombia-Medellin, Iraq, Israel, Japan, New Zealand, Northern Ireland, PRC - Shenzhen, Romania, Saudi Arabia, South Africa and Spain-Murcia did not have an age restricted Part 2 sample. All other surveys, with the exception of Nigeria and Ukraine (which were age restricted to ≤ 39) were age restricted to ≤ 44.

*^e^* The response rate is calculated as the ratio of the number of households in which an interview was completed to the number of households originally sampled, excluding from the denominator households known not to be eligible either because of being vacant at the time of initial contact or because the residents were unable to speak the designated languages of the survey. The weighted average response rate is 69.5%.

*^f^* Colombia moved from the "lower and lower-middle income" to the "upper-middle income" category between 2003 (when the Colombian National Study of Mental Health was conducted) and 2010 (when the Medellin Mental Health Household Study was conducted), hence Colombia's appearance in both income categories. For more information, please see footnote *a*.

*^g^* People’s Republic of China

*^h^* For the purposes of cross-national comparisons we limit the sample to those 18+.

**Supplemental table 2. Sociodemographic and MDD characteristics of the sample.**

|  | **Respondents without 12-month MDD**  **(N = 73,506)** | | **Respondents with 12-month MDD**  **(N = 6,684)** | |
| --- | --- | --- | --- | --- |
|  | % / mean | SE | % / mean | SE |
| ***Sociodemographics*** |  |  |  |  |
| Age (mean) | 42.5 | 0.1 | 41.2 | 0.3 |
| Sex (% female) | 51.1 | 0.3 | 66.8 | 0.8 |
| Education status (%) |  |  |  |  |
| Low | 17.0 | 0.2 | 18.0 | 0.6 |
| Low-average | 24.7 | 0.2 | 28.1 | 0.8 |
| High-average | 36.3 | 0.3 | 32.5 | 0.8 |
| High | 22.0 | 0.3 | 21.4 | 0.7 |
| Marital status (%) |  |  |  |  |
| Currently married | 62.6 | 0.3 | 51.5 | 0.8 |
| Previously married | 11.6 | 0.2 | 20.6 | 0.6 |
| Never married | 25.8 | 0.3 | 27.8 | 0.8 |
| Employment status (%) |  |  |  |  |
| Employed | 59.3 | 0.3 | 53.7 | 0.9 |
| Student | 4.7 | 0.1 | 4.7 | 0.4 |
| Homemaker | 11.8 | 0.2 | 14.7 | 0.6 |
| Retired | 13.5 | 0.2 | 10.9 | 0.5 |
| Other | 10.7 | 0.2 | 15.9 | 0.6 |
| Income status (%) |  |  |  |  |
| Low | 27.0 | 0.3 | 31.1 | 0.8 |
| Low-average | 23.5 | 0.2 | 25.3 | 0.7 |
| High-average | 26.1 | 0.2 | 24.3 | 0.7 |
| High | 23.4 | 0.3 | 19.2 | 0.7 |
| ***MDD characteristics*** |  |  |  |  |
| Chronic MDD (%) |  |  | 34.4 | 0.8 |
| Worst QIDS in past 12 months (mean)^a^ |  |  | 15.1 | 0.1 |
| Minimal symptoms (QIDS < 6) (%) |  |  | 2.2 | 0.3 |
| Mild symptoms (QIDS 6 - 10) (%) |  |  | 12.6 | 0.7 |
| Moderate symptoms (QIDS 11 - 15) (%) |  |  | 36.0 | 0.9 |
| Severe symptoms (QIDS 16 - 20) (%) |  |  | 34.5 | 0.9 |
| Very severe symptoms (QIDS > 20) (%) |  |  | 14.7 | 0.7 |
| Age of MDD onset |  |  |  |  |
| MDD onset before 18 years old (%) |  |  | 27.8 | 0.7 |
| MDD onset between age 18 and 34 (%) |  |  | 42.4 | 0.8 |
| MDD onset between age 35 and 49 (%) |  |  | 19.7 | 0.7 |
| MDD onset between age 50 and 65 (%) |  |  | 7.8 | 0.4 |
| MDD onset at age 65 or later (%) |  |  | 2.3 | 0.2 |

MDD=major depressive disorder, SE=standard error, QIDS=Quick Inventory of Depressive Symptoms

^a^  The QIDS was available for 4760 participants, as it was not assessed in ESEMED surveys (Netherlands, Belgium, France, Italy, Spain, and Germany), Shenzhen, and South Africa (1357 participants), and an additional 567 participants had missing data.

**Supplemental Table 3. Association between previous disorders and days out of role.**

|  | **Beta** | **95% CI** | **p** |
| --- | --- | --- | --- |
| *Model 1* |  |  |  |
| No previous disorders (ref) | 0.0 | - | - |
| Depressive distress disorders | 10.9 | 1.1-20.8 | 0.030 |
| Non-depressive distress disorders | 16.6 | 8.4-24.8 | <0.001 |
| Fear disorders | 13.8 | 6.4-21.3 | <0.001 |
| Externalizing disorders | 5.1 | -2.5-12.6 | 0.19 |
| *Model 2* |  |  |  |
| No previous disorders (ref) | 0.0 | - | - |
| Depressive distress disorders | 9.3 | -0.3-18.8 | 0.057 |
| Non-depressive distress disorders | 14.3 | 5.8-22.8 | <0.001 |
| Fear disorders | 15.7 | 8.2-23.1 | <0.001 |
| Externalizing disorders | 6.7 | -1.2-14.6 | 0.10 |
| *Model 3* |  |  |  |
| No previous disorders (ref) | 0.0 | - | - |
| Depressive distress disorders | 6.3 | -2.5-15.1 | 0.16 |
| Non-depressive distress disorders | 13.2 | 4.9-21.4 | 0.002 |
| Fear disorders | 14.1 | 6.8-21.4 | <0.001 |
| Externalizing disorders | 4.9 | -2.9-12.6 | 0.22 |

*Association between days out of role and previous depressive distress, non-depressive distress, fear, or externalizing disorders, among respondents with 12-month MDD.*

MDD=major depressive disorder; CI=confidence interval

Depressive distress disorders: major depressive disorder and dysthymia; non-depressive distress disorders: generalized anxiety disorder and post-traumatic stress disorder; fear disorders: agoraphobia, panic disorder, separation anxiety disorder, social anxiety disorder, specific phobia; externalizing disorders: intermittent explosive disorder, attention deficit hyperactivity disorder, conduct disorder, oppositional defiant disorder, alcohol abuse, alcohol dependence, drug abuse, and drug dependence

Model 1: Adjusted for country

Model 2: Adjusted for country, age, sex

Model 3: Adjusted for country, age, sex, education, marital status, and employment status

**Supplemental table 4. Association between previous disorders and severe role impairment, after additional adjustment for MDD characteristics.**

|  | **Home** | | | **Work** | | | **Relationship** | | | **Social** | | | **Any** | | |
| --- | --- | --- | --- | --- | --- | --- | --- | --- | --- | --- | --- | --- | --- | --- | --- |
|  | OR | 95% CI | p | OR | 95% CI | p | OR | 95% CI | p | OR | 95% CI | p | OR | 95% CI | p |
| No previous disorders (ref) | - | - | - | - | - | - | - | - | - | - | - | - | - | - | - |
| Depressive distress disorders | 0.4 | 0.3-0.6 | <0.001 | 0.5 | 0.4-0.7 | <0.001 | 0.7 | 0.5-1.0 | 0.054 | 0.6 | 0.4-0.8 | 0.001 | 0.6 | 0.4-0.8 | 0.002 |
| Non-depressive distress disorders | 1.3 | 1.0-1.5 | 0.019 | 1.3 | 1.0-1.6 | 0.022 | 1.4 | 1.2-1.7 | <0.001 | 1.3 | 1.1-1.6 | 0.003 | 1.3 | 1.1-1.6 | 0.005 |
| Fear disorders | 0.9 | 0.8-1.1 | 0.46 | 1.0 | 0.8-1.2 | 0.66 | 1.1 | 0.9-1.4 | 0.16 | 1.2 | 1.0-1.5 | 0.029 | 1.2 | 1.0-1.4 | 0.12 |
| Externalizing disorders | 1.1 | 0.9-1.4 | 0.31 | 1.1 | 0.9-1.4 | 0.30 | 1.2 | 1.0-1.5 | 0.042 | 1.4 | 1.1-1.7 | 0.001 | 1.4 | 1.2-1.8 | 0.001 |
| **MDD characteristics** |  |  |  |  |  |  |  |  |  |  |  |  |  |  |  |
| MDD age of onset (in 10 years) | 1.1 | 1.0-1.1 | 0.08 | 1.2 | 1.1-1.3 | <0.001 | 1.1 | 1.0-1.2 | 0.19 | 1.1 | 1.0-1.2 | 0.023 | 1.1 | 1.0-1.2 | 0.022 |
| MDD chronicity (lifetime) | 1.2 | 1.0-1.4 | 0.11 | 1.0 | 0.9-1.3 | 0.68 | 0.9 | 0.8-1.1 | 0.42 | 0.8 | 0.7-1.0 | 0.102 | 1.1 | 0.9-1.4 | 0.30 |
| MDD severity (12-month) | 1.2 | 1.2-1.3 | <0.001 | 1.2 | 1.2-1.2 | <0.001 | 1.2 | 1.2-1.2 | <0.001 | 1.2 | 1.2-1.2 | <0.001 | 1.2 | 1.2-1.3 | <0.001 |

*Association between severe role impairment and previous depressive distress, non-depressive distress, fear, or externalizing disorders, among respondents with 12-month MDD and additionally adjusted for MDD characteristics*

MDD=major depressive disorder; OR=odds ratio; CI=confidence interval

Depressive distress disorders: major depressive disorder and dysthymia; non-depressive distress disorders: generalized anxiety disorder and post-traumatic stress disorder; Fear disorders: agoraphobia, panic disorder, separation anxiety disorder, social anxiety disorder, specific phobia; externalizing disorders: Intermittent explosive disorder, attention deficit hyperactivity disorder, conduct disorder, oppositional defiant disorder, alcohol abuse, alcohol dependence, drug abuse, and drug dependence

Analyses adjusted for country, age, sex, education, marital status, employment status, and MDD characteristics

**Supplemental Table 5. Association between previous disorders and days out of role, after adjustment for MDD characteristics.**

|  | **Beta** | **95% CI** | **p** |
| --- | --- | --- | --- |
| No previous disorders (ref) | - | - | - |
| Depressive distress disorders | 6.0 | -3.9-15.8 | 0.24 |
| Non-depressive distress disorders | 6.6 | -1.5-14.7 | 0.11 |
| Fear disorders | 7.2 | 0.0-14.4 | 0.049 |
| Externalizing disorders | 2.3 | -5.0-9.7 | 0.54 |
| **MDD characteristics** |  |  |  |
| MDD age of onset (in 10 years) | 3.0 | -0.9-6.8 | 0.13 |
| MDD chronicity (lifetime) | 19.8 | 12.0-27.6 | <0.001 |
| MDD severity (12-month) | 4.2 | 3.3-5.0 | <0.001 |

*Association between days out of role and previous depressive distress, non-depressive distress, fear, or externalizing disorders, among respondents with 12-month MDD, after additional adjustment for MDD characteristics.*

MDD=major depressive disorder; CI=confidence interval

Depressive distress disorders: major depressive disorder and dysthymia; non-depressive distress disorders: generalized anxiety disorder and post-traumatic stress disorder; fear disorders: agoraphobia, panic disorder, separation anxiety disorder, social anxiety disorder, specific phobia; externalizing disorders: intermittent explosive disorder, attention deficit hyperactivity disorder, conduct disorder, oppositional defiant disorder, alcohol abuse, alcohol dependence, drug abuse, and drug dependence

Analyses adjusted for country, age, sex, education, marital status, employment status, and MDD characteristics

**Supplemental table 6. Association between previous disorders and suicidality after additional adjustment for MDD characteristic.**

|  | **Ideation** | | | **Plan** | | | **Attempt** | | |
| --- | --- | --- | --- | --- | --- | --- | --- | --- | --- |
|  | OR | 95% CI | p | OR | 95% CI | p | OR | 95% CI | p |
| No previous disorders (ref) | - | - | - | - | - | - | - | - | - |
| Depressive distress disorders | 0.8 | 0.5-1.3 | 0.29 | 1.0 | 0.5-2.0 | 0.98 | 1.6 | 0.7-3.9 | 0.30 |
| Non-depressive distress disorders | 1.1 | 0.9-1.4 | 0.33 | 1.0 | 0.7-1.5 | 0.94 | 1.1 | 0.7-1.7 | 0.72 |
| Fear disorders | 1.4 | 1.1-1.7 | 0.006 | 1.4 | 1.0-2.0 | 0.041 | 1.7 | 1.1-2.6 | 0.020 |
| Externalizing disorders | 1.6 | 1.3-2.0 | <0.001 | 2.1 | 1.5-2.9 | <.001 | 1.6 | 1.0-2.6 | 0.030 |
| **MDD characteristics** |  |  |  |  |  |  |  |  |  |
| MDD age of onset (in 10 years) | 0.9 | 0.8-1.0 | 0.027 | 1.0 | 0.8-1.1 | 0.70 | 1.1 | 0.9-1.3 | 0.58 |
| MDD chronicity (lifetime) | 0.9 | 0.7-1.2 | 0.43 | 1.0 | 0.7-1.4 | 0.95 | 1.1 | 0.7-1.8 | 0.66 |
| MDD severity (12-month) | 1.2 | 1.2-1.2 | <0.001 | 1.2 | 1.1-1.2 | <0.001 | 1.2 | 1.1-1.3 | <0.001 |

*Suicidality for respondents with 12-month MDD with previous depressive distress, non-depressive distress, fear, or externalizing disorders, vs. those without any previous disorders, additionally adjusted for MDD characteristics*

MDD=major depressive disorder; OR=odds ratio; CI=confidence interval

Depressive distress disorders: major depressive disorder and dysthymia; non-depressive distress disorders: generalized anxiety disorder and post-traumatic stress disorder; fear disorders: agoraphobia, panic disorder, separation anxiety disorder, social anxiety disorder, specific phobia; externalizing disorders: Intermittent explosive disorder, attention deficit hyperactivity disorder, conduct disorder, oppositional defiant disorder, alcohol abuse, alcohol dependence, drug abuse, and drug dependence

Adjusted for country, age, sex, education, marital status, employment status, and MDD characteristics

**Supplemental Table 7. Association between previous disorders, divided into remitted or current comorbid disorders, and days out of role.**

|  | **Beta** | **95% CI** | **p** |
| --- | --- | --- | --- |
| No previous disorders (ref) | - | - | - |
| Depressive distress disorders | 5.4 | -4.2-14.9 | 0.27 |
| Non-depressive distress disorders, remitted | -1.3 | -11.9-9.3 | 0.81 |
| Non-depressive distress disorders, current | 8.5 | -0.6-17.5 | 0.066 |
| Fear disorders, remitted | -4.8 | -13.0-3.4 | 0.25 |
| Fear disorders, current | 10.8 | 2.8-18.8 | 0.008 |
| Externalizing disorders, remitted | -2.8 | -10.4-4.9 | 0.48 |
| Externalizing disorders, current | 7.5 | -3.7-18.8 | 0.19 |

*Days out of role for respondents with 12-month MDD with previous disorders divided in remitted or current episodes, vs. those without any previous disorder*

MDD=major depressive disorder; CI=confidence interval

Depressive distress disorders: major depressive disorder and dysthymia; non-depressive distress disorders: generalized anxiety disorder and post-traumatic stress disorder; fear disorders: agoraphobia, panic disorder, separation anxiety disorder, social anxiety disorder, specific phobia; externalizing disorders: intermittent explosive disorder, attention deficit hyperactivity disorder, conduct disorder, oppositional defiant disorder, alcohol abuse, alcohol dependence, drug abuse, and drug dependence

Analyses adjusted for country, age, sex, education, marital status, employment status, and MDD characteristics (age of onset, chronicity, severity)
